# Portable Homemade Magnetic Hyperthermia Apparatus: Preliminary Results

**DOI:** 10.3390/nano14221848

**Published:** 2024-11-19

**Authors:** Teresa Castelo-Grande, Paulo A. Augusto, Lobinho Gomes, Eduardo Calvo, Domingos Barbosa

**Affiliations:** 1 LEPABE—Laboratory for Process Engineering, Environment, Biotechnology and Energy, Faculty of Engineering, University of Porto, Rua Dr. Roberto Frias, 4200-465 Porto, Portugal; dbarbosa@fe.up.pt; 2 AliCE—Associate Laboratory in Chemical Engineering, Faculty of Engineering, University of Porto, Rua Dr. Roberto Frias, 4200-465 Porto, Portugal; 3 Institute of Molecular and Cellular Biology of Cancer, CSIC/University of Salamanca (GIR Citómica), 37007 Salamanca, Spain; pauloaugusto@usal.es; 4 CEADIR—Center for Environmental Studies and Rural Revitalization, Avenida Filiberto Villalobos, 119, 37007 Salamanca, Spain; 5 Faculty of Natural Sciences, Engineering and Technologies, Lusófona University of Porto, R. de Augusto Rosa 24, 4000-098 Porto, Portugal; lobinho.gomes@sapo.pt; 6 CEFT—Center of Study of Phenomena’s of Transport, Faculty of Engineering, University of Porto, Rua Dr. Roberto Frias, 4200-465 Porto, Portugal; ecalvo@edu.fe.up.pt

**Keywords:** magnetic hyperthermia, cancer treatment, nanotechnologies, nanomaterials, portable apparatus

## Abstract

This study aims to describe and evaluate the performance of a new device for magnetic hyperthermia that can produce an alternating magnetic field with adjustable frequency without the need to change capacitors from the resonant bank, as required by other commercial devices. This innovation, among others, is based on using a capacitator bank that dynamically adjusts the frequency. To validate the novel system, a series of experiments were conducted using commercial magnetic nanoparticles (MNPs) demonstrating the device’s effectiveness and allowing us to identify new challenges associated with the design of more powerful devices. A computational model was also used to validate the device and to allow us to determine the best system configuration. The results obtained are consistent with those from other studies using the same MNPs but with magnetic hyperthermia commercial equipment, confirming the good performance of the developed device (e.g., consistent SAR values between 1.37 and 10.80 W/gMNP were obtained, and experiments reaching temperatures above 43 °C were also obtained). This equipment offers additional advantages, including being economical, user-friendly, and portable.

## 1. Introduction

Over the last twenty years, magnetic hyperthermia (MH) has been studied as a potential method for treating cancer. It is undergoing clinical trials in Germany [1] and is also starting to be tested clinically in other countries [2]. It is an interdisciplinary field that demands knowledge integration from diverse areas such as quantum physics, materials research, electrical engineering, chemical engineering, biology, biochemistry, and medicine. As a result, scientists face substantial challenges in fully comprehending this technique and its associated therapies, for which further exploration is thus needed.

Hyperthermia treatment, involving heat generation, holds immense potential. For cancer therapy, it entails raising the local temperature of the tumor, thus modifying the physiology of the diseased cells and ultimately causing tissue necrosis. This treatment can effectively complement existing cancer therapies, including chemotherapy, radiotherapy, surgery, and immunotherapy.

Hyperthermia treatments can be categorized into different types based on the extent of the temperature increase:

(1) High hyperthermia reaches temperatures above 46 °C (up to 56 °C), causing direct tissue necrosis, coagulation, or carbonization of cells.
(2) Moderate hyperthermia (41 °C < T < 46 °C) has various effects at the cellular and tissue levels. In this temperature range, cells undergo heat stress, resulting in the activation and/or initiation of many extracellular degradation mechanisms, such as protein denaturation, protein folding, DNA aggregation, and cross-linking.
(3) Low hyperthermia uses temperatures below 41 °C and is applied to treat rheumatic diseases in physiotherapy. It is important to notice that the effectiveness of any hyperthermia treatment depends significantly on the temperatures reached in the targeted sites, the duration of exposure, and the specific characteristics of the cancer cells [3]. The National Cancer Institute (NCI) recognizes three different types of hyperthermia, which are classified based on the area where it is applied and the extent of the area to be treated: local, regional, and whole-body hyperthermia. In local hyperthermia, the primary objective is to heat only tumor cells without damaging healthy tissues. Local hyperthermia is currently a major focus due to its ability to target heat within a specified region [4].

Traditionally, hyperthermia treatment was administered through external devices that transfer energy to the tissues via irradiation with light or electromagnetic waves. Today, there are various techniques available for inducing hyperthermia, such as ultrasound [5], radio frequencies [6], microwave range waves [7], infrared radiation [8], nanoparticle-assisted photothermal therapy [9,10,11], and the use of magnetically excitable nanoparticles. However, each of these methods has its own limitations. Oncologists usually combine hyperthermia treatment with radiotherapy, chemotherapy, or both [12]. Some of the challenges in traditional hyperthermia treatment include:

(1) The inevitable heating of healthy tissue, resulting in burns, blisters, and discomfort;
(2) Limited penetration of heat into body tissues by microwave, laser, and ultrasound energy;
(3) Thermal underdosing in the target area, particularly in areas protected by pelvic or nape bones, often leads to recurrent tumor growth, remaining largely unresolved.

The potential use of magnetic materials in cancer treatment through hyperthermia was initially suggested in 1957, aiming to convert magnetic energy into thermal energy [13]. The rapid progress in magnetic nanoparticles (MNPs) synthesis has represented significant advances in this type of hyperthermia. Hyperthermia treatment using MNPs presents multiple advantages over traditional hyperthermia treatment, as outlined in Table 1.

The exploration of new magnetic nanoparticles is currently at the forefront of biomedical research, particularly in magnetic hyperthermia for cancer treatment. Extensive research is being conducted on superparamagnetic nanoparticles, with an average diameter (σ) of just a few tens of nanometers, to assess their potential in this area. In magnetic hyperthermia, these nanoparticles are exposed to an alternating magnetic field (AMF) with high amplitude ($H_{0}$) and high-frequency ($f_{0}$).

The “tunable” magnetic properties of MNPs are essential for their biomedical applications. These properties, such as magnetic susceptibility (χ), blocking temperature ($T_{B}$), relaxation time (τ), and saturation magnetization ($M_{s}$), can be tailored through different synthesis processes to create specific MNPs for magnetic hyperthermia treatments. The specific absorption rate (SAR), proportional to the nanoparticles’ concentration, is a key indicator of energy dissipation as heat per unit of mass and can be calculated using the initial slope method.

Some researchers prefer to calculate the specific loss power (SLP) instead of the SAR, as its definition is less ambiguous. Many studies have measured the SLP for different MNPs. The heating generated by MNPs is attributed to various factors such as eddy currents, the Brownian effect (Brownian relaxation), the crossing of anisotropic barriers (Néel relaxation), and magnetic energy losses due to the magnetic hysteresis of the MNPs.

Utilizing the linear response theory (LRT), valid for low-applied fields and/or highly anisotropic magnetic nanoparticles, this heating power can be mathematically expressed as detailed by [18].

(1) $$SLP=\pi \times \mu_{0}\times \chi_{0}\times H_{0}^{2}\times \frac{2\times f_{0}\times \tau }{\left(1+{\left(2\times \pi \times f_{0}\times \tau \right)}^{2}\right)}\left(\frac{W}{g}\right)\;\;\;\;\;\;\;\;$$

where $SLP,\chi_{0},\;H_{0},\mu_{0}$ and $f_{0}$ represent specific loss power or specific power absorption, static susceptibility, magnetic field amplitude, vacuum permeability, and frequency, respectively, and τ is the relaxation time, as detailed in [19,20]. Equation (1) demonstrates that both the field amplitude and field frequency influence the dissipated power of MNPs.

Before conducting in vivo experiments, the MNPs must undergo thorough preliminary studies in chemical laboratories to characterize their physical and chemical properties. In addition, in vitro experiments in cellular biology laboratories are necessary to evaluate heating effects and the extent of necrosis or apoptosis in cell cultures. A compact experimental setup, such as the device developed in our lab, provides an excellent alternative for conducting rapid trials and measurements in all these laboratories, making it a powerful tool.

The main goal for synthesized MNPs is to produce a high SAR with a low concentration of particles when exposed to an alternating magnetic field. There are various systems proposed for generating alternating magnetic fields, including single- and double-layer solenoids, Helmholtz coils, inductors with a ferromagnetic core (in horseshoe shape), and coils made with Litz wire [21,22]. The single-layer solenoid refrigerated system is the most widely used, even in commercial equipment, and it is the primary focus of this work.

The development of MH equipment remains vastly unexplored due to its intricate technology demands, necessitating both high power and high frequency at the same time. Commercially available devices are, therefore, complex equipment that also does not present the desired compactness and portability. Consequently, and due to its complexity and, hence, associated costs only a handful of companies manufacture this equipment, leading to significant economic barriers that, among others, impede more in-depth research and widespread application of this promising technique.

It is already known that in order to create devices that are able to achieve the frequencies and magnetic fields required for magnetic hyperthermia, the best option is to use a resonant circuit. Many different systems with different configurations have been developed so far to reach this goal. The current options that exist commercially are complicated (thus hard to adapt and costly) and/or do not allow for a dynamic change of frequencies, making the overall device rather complex, expensive, hard to handle, and non-portable. In the last years, research has been performed trying to innovate and achieve devices where a change of frequencies does not require the physical change of capacitors, among other concerns; however, all these solutions present limited success only. For example, Mazon et al. [23] present a device where frequency tuning of a magnetic hyperthermia device is achieved by switching control capacitors. Nonetheless, these and similar cases present the problem that the tactic used for frequency tunning implies the use of microprocessors with a drive circuit and an H bridge and an inverter, demanding also the use of a PLL circuit for better enhancing the results of the output frequencies, making the all-device complex, costly, and non-portable. In the work that is detailed in this article, we present an innovative system that was very challenging to design and develop, as it joined the capacity of frequency tuning by switching control capacitors with the simplified version of a resonant circuit, without the need to use complex microprocessors, inverters, etc. The outcome is a device capable of performing magnetic hyperthermia with dynamical frequency tunning based on a simplified circuit, which makes it low-cost, portable, and flexible, and, thus, allows for simpler and cheaper magnetic hyperthermia research studies and also for possible worldwide commercial use.

## 2. Materials and Methods

### 2.1. Magnetic Field Simulations

The magnetic field simulations were conducted using the “Magnetic fields” module from the commercial software COMSOL Multiphysics^®^, version 6.0. For computational purposes, an HP workstation Z4 (HP Headquarters, Lisbon, Portugal) with an Intel(R) Xeon(R) W-2295 CPU @ 3.00 GHz was used.

**Figure 1:**
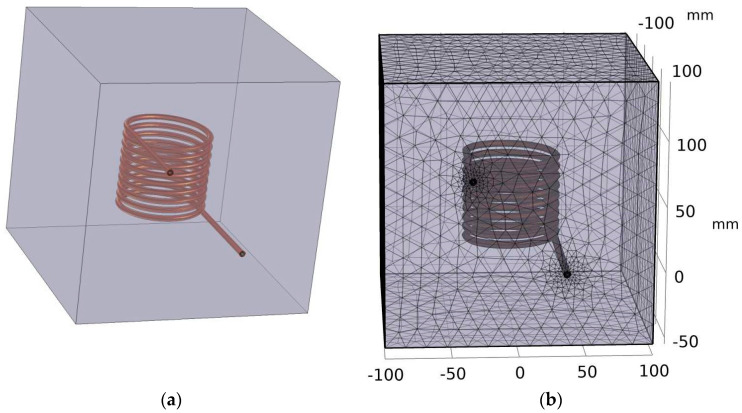
Three-dimensional view of solenoid S5. (**a**) The copper coil contains interior water for cooling inside, while the surrounding domain contains air (**b**) computational mesh.

A typical solenoid shape was chosen because it preserves the direction of the magnetic field within its interior [24]. The coil itself is composed of hollow copper tubes with an inner radius of 1.5 mm and thickness of 1 mm, into which a water pumping system is connected to control temperature changes due to the Joule effect. To complete the geometry step, the coil was placed inside a parallelepiped domain composed of air. The example of S5 is depicted in Figure 1. The simulation of the magnetic field was performed with a finite element method (FEM), where the domain is discretized into small triangular elements. In each element, the magnetic field is computed through a frequency domain solver. In our simulation, we employed a dense mesh to accurately capture the system’s complexities. For the copper tube and the water domain, we implemented a specialized boundary condition designed to effectively simulate the skin depth effect associated with the electric current. This approach allowed us to better represent the penetration of the current into the conductive material, ensuring a more realistic analysis of the interaction between the copper tube and the surrounding water. In total, the mesh consists of 365,281 elements with a minimum relative quality of 0.178 and an average quality of 0.6485, being totally composed of 3 main domains (water, coil, air), 18 faces, 36 edges, and, finally, by 24 points.

Magnetostatic numerical models:

(2) ∇×H=J

(3) $$B=\mu_{r}\mu_{0}H$$

(4) B=∇×A

where ***J*** is the current density vector, given by the product of the material’s electrical conductivity (σ) and the electric field (E), J=σ E. This field is computed based on the type of excitation in the coil. In this study, the coils are voltage-driven, so the electric field is derived from the divergence of the electric potential, E=−∇·V. To account for the Joule effect, the electrical losses are calculated using the following equation:

(5) $$Q=\frac{1}{T}\int_{t}^{t+T}J\cdot E\;dt$$

where Q represents the electromagnetic losses. Finally, magnetic insulation boundary conditions, $\hat{n}\times A$ were applied to the faces of the air parallelepiped domain. The materials were selected from the “*built-in*” libraries from the COMSOL software. The properties are listed in Table 2.

### 2.2. Hyperthermia Studies

The initial examination of the developed magnetic hyperthermia (MH) system as presented in this article involved the use of commercially available MNPs. These MNPs were carefully chosen based on a comprehensive review of the literature, highlighting their positive results [25,26,27,28,29]. Their characteristics and designations are detailed in Table 3. Hence, seven different iron-oxide-based magnetic liquid samples from Chemicell GmbH (Berlin, Germany) were evaluated (Table 4), following studies by Kallumadil et al. [26]); Yaremenko, A.V et al. [30]; and Wang, Huang et al. [31]. These samples varied in size and core-shell configurations, all featuring a magnetite core. The MNPs had previously undergone comprehensive testing and characterization, particularly concerning their hydrodynamic diameters.

**Figure 2:**
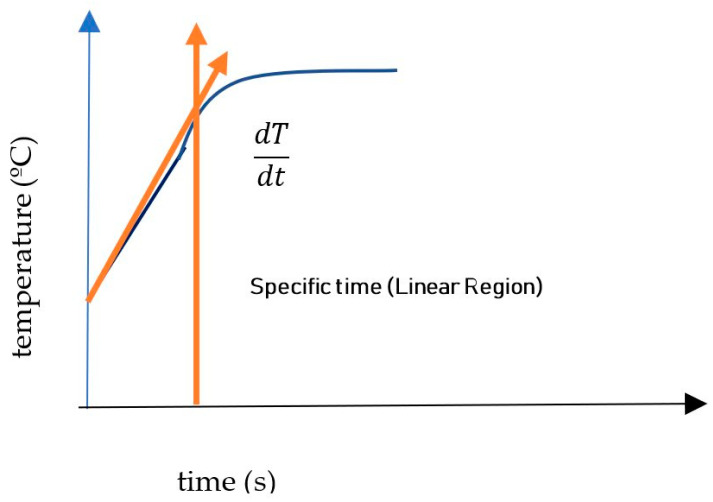
Initial slope method (ISM) for calculating the specific absorption rate (SAR).

The heating efficacy is represented by the specific absorption rate (SAR) value, expressed in W/g_(Magnetic NanoParticles)_. This value corresponds to the amount of energy converted into heat (J) per unit time (s) and mass (m_mag_) of the magnetic material. The SAR was calculated using the initial slope method (Figure 2), which considers the first few minutes (Equation (6)) within the linear variation range of the heating curve (adiabatic regime). This period is typically set at 100 s based on theoretical assumptions (low frequency and magnetic field).

(6) $${S.A.R}_{\left(\mathrm{Specific}\;\mathrm{Absortion}\;\mathrm{Rate}\right)}=C_{\mathrm{msol}}\times \frac{m_{sol}}{m_{mag}}\frac{dT}{dt}=\frac{(m_{H_{2O}\times Cp_{H_{2O}+}\;m_{MNP\;}\times Cp_{MNP)}}}{\;m_{MNP\;}}\times \frac{dT}{dt}$$

where $C_{\mathrm{msol}}$ is the specific heat of the solution, $m_{sol}\;$is the mass of the solution, $m_{mag}$ is the mass of the MNPs, and $\frac{dT}{dt}$ is the maximum value of the initial linear slope.

The concentration range for measuring the specific absorption rate (SAR) of the of the MNPs was 5 to 25 mg_MNP_/mL. This measurement was conducted using the developed apparatus by determining the heating curve (temperature variation over time). For statistical accuracy, three measurements were taken for each sample.

In Appendix A, details are shown about how magnetic fields were measured and accounted for.

## 3. Results and Discussion

### 3.1. Configuration, Specifications, and Fundamental Operational Principles of an Alternating Magnetic Field Generator

In the past two decades, there has been remarkable progress in the development of devices for magnetic hyperthermia applications. These devices utilize alternating magnetic fields at high frequencies and power levels [33]. However, most of these devices are primarily designed for whole-body applications [1]. Due to their large size, these devices are not suitable for use in research laboratories. Recently, various switched-mode resonant inverters have been developed for medical use in electromagnetic thermotherapy. These inverters use voltage-fed high-frequency applicators with power metal-oxide-semiconductor field-effect transistors (MOSFETs) operating based on resonant circuits as further explained below [34]. To achieve the necessary alternating magnetic field for hyperthermia, a high alternating electric current at a specific frequency must be passed through a coil, typically at high frequencies (>100 kHz). To accomplish this, two main technologies are commonly used: resonant circuits and H-bridge-based circuits, also known as inverters. For more information on how these circuits work and their components, see Cabrera et al., 2019 [35].

**Figure 3:**
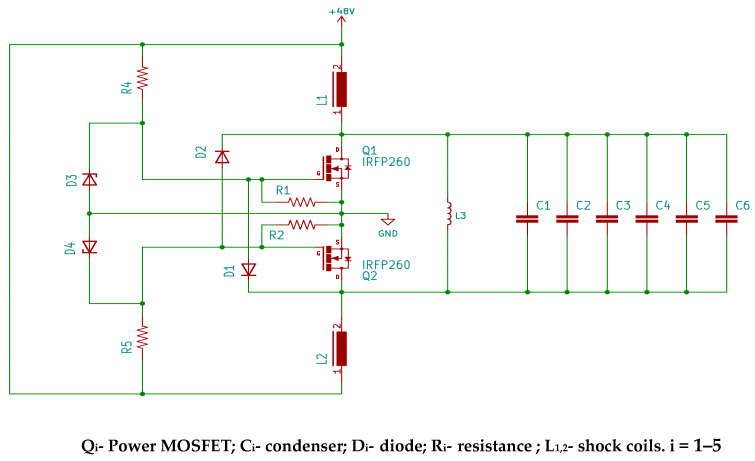
Parallel LC resonant circuit.

In this groundbreaking work, a resonant circuit was employed, harnessing a technique that has undergone extensive study over the past three decades. This approach has captured substantial attention from both academic and industrial research communities due to its remarkable characteristics: smooth waveforms, exceptional efficiency, and high-power density. Resonant circuits are based on transforming a constant direct current power source into a sinusoidal wave. This is made possible through a resonant circuit based on a parallel LC (inductor/capacitor) configuration, as depicted in Figure 3.

Resonant converters encompass a diverse and extensive family, which can pose a straightforward description challenge. However, one common feature shared by most, if not all, is their reliance on a “resonant inverter” to convert DC voltage into sinusoidal voltage and deliver AC power. These converters utilize major topologies such as full-bridge zero voltage switching (ZVS), half-bridge ZVS, full-bridge zero current switching (ZCS), and half-bridge ZCS series converters [36]. For this project, we opted for the half-bridge ZVS series converter, a simple yet widely used choice (see Figure 1). The ZVS system is typically employed in high-frequency switching applications, offering advantages including reduced power losses, enhanced reliability, and improved overall performance of power electronic systems [37].

To attain maximum performance, the converter needs to operate at the resonant frequency. Equations (7) and (8) precisely define the resonance frequency f_r_ and the peak voltage V_M_ across the coil for the basic ZVS series converter at the operating frequency.

(7) $$f_{r}=\frac{1}{2\times \pi \times \sqrt{L\times C}}$$

(8) $$V_{M}=X_{L}\times I_{max}=2\times \pi \times f_{r}\times L\times I_{max}$$

In Equations (7) and (8), L denotes the coil’s inductance, while *C* represents the system’s capacitance. *XL* stands for inductive reactance, which varies with frequency.

In our research, we attempted to use the simplest and most efficient system that would allow us to construct an economical and portable device for use by the scientific community.

**Figure 4:**
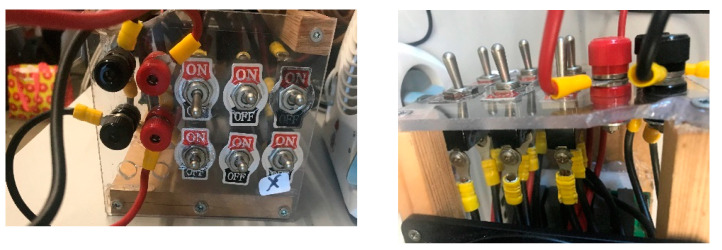
Image of the switch set that controls the capacitor bank.

The experimental program initiated with the establishment of a resonant circuit to generate a sinusoidal wave. The wave’s frequency is contingent on the coil configuration and the capacitors positioned in parallel to form the resonant oscillator circuit. Typically, this circuit maintains a fixed frequency, obstructing the analysis of particle behavior at different frequencies for identifying the optimal operating value. To surmount this limitation, a bank of capacitors, managed by a set of switches (six in this scenario), was introduced into the circuit. These switches enable the parallel placement of more or fewer capacitors with the test coil, thereby modifying the resonant frequency of the LC circuit and consequently adjusting the test frequency (see Figure 4). This apparatus has demonstrated remarkable effectiveness in operating at high powers and high frequencies (frequency range 69–303 khz and generated magnetic field range 4 mT–16 mT).

**Figure 5:**
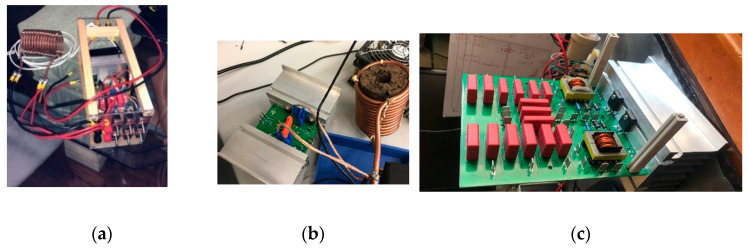
(**a**) Initial prototype of our system, (**b**) the system being currently applied, (**c**) a new and more powerful prototype system (resonant) that is being tested.

Upon testing our initial resonant system (see Figure 5a) with a fixed bank of capacitors, we achieved notable positive results. These results were particularly evident when we modified the coil’s configuration to increase field concentration by adjusting the current flow due to lower impedance (2.5 to 5 µH inductance) and by changing the frequency.

In Figure 5, the progression from our initial system prototype to the current development stage is depicted, even into a new, more powerful prototype currently under testing. The magnetic hyperthermia device assembly consists of five key components:

(1) Power source (VELLEMAN, Model: LABPS6030SM);
(2) Electric circuit (designed by us and manufactured by JMP Electronics (Jinhu, China));
(3) Cooling system (designed by us);
(4) Oscilloscope coupled with a probe for measuring the magnetic field (see Appendix A) and verifying the waveform passing through the coil (Promax Electronics, Model: OD-624);
(5) Temperature recorder and probe for measuring the sample temperature (Chaviun1822 with probe K presenting an accuracy ±2%). In the magnitude range of the magnetic field, the inference is minimal, as demonstrated in the next section [32,33,38].

The power source was carefully chosen to ensure the required voltage (ΔV) for generating an electric current capable of creating a high-intensity magnetic field. The specific MOSFET selected, IRFP260, can handle a maximum current of 60 A and support a voltage of 200 V.

The resonant system and coil were meticulously designed to produce a uniform magnetic field inside the sample. Several coils were designed and tested, the default system being composed of a coil, made of copper and comprising a 2 cm diameter, 11 cm length, and 17 turns, designed to be cooled by flowing water to maintain room temperature. It is important to notice that under ideal conditions, Equations (7) and (8) are well fitted to the real maximum current and frequency of the resonant circuit, respectively.

The maximum intensity of the magnetic field in the coils is given by Equation (9),

(9) $$B_{MAX}=\frac{\mu_{0}\times I_{curr.max}\times N}{l_{e}}$$

**Figure 6:**
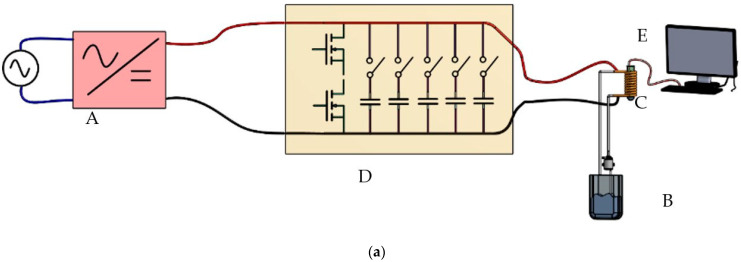

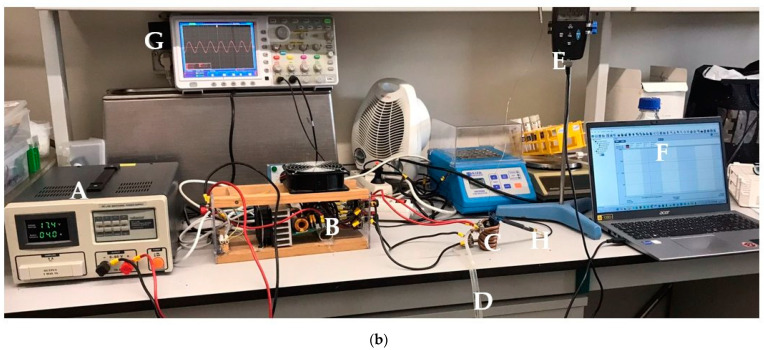
Overview of the system (**a**) and photographs of the apparatus and of the system (**b**): A—power source; B—system; C—solenoid; D—cooling system; E—equipment for measuring temperature; F—computer for acquiring and registering the temperature; G and H—oscilloscope coupled with a probe for measuring the magnetic field and verifying the waveform passing through the coil [39].

**Figure 7:**
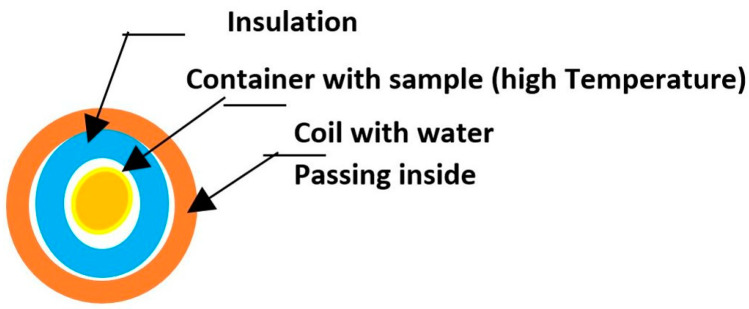
Insulating system.

*N* represents the number of turns of the coil, *μ*_0_ stands for the magnetic permeability in a vacuum (4π × 10^−7^ H/m), *I_curr.max_* denotes the maximum current intensity, and *l_e_* indicates the length of the solenoid. The high current running through the system causes the solenoid to heat up due to the Joule effect. To maintain a constant temperature, it is essential to cool it by circulating water within the solenoid. Priority is given to improve the insulation system, and currently, we are exploring materials such as mineral wool, AMORIM-expanded cork, and Styrofoam for the sample holder and insulation. Figure 6 and Figure 7 illustrate a diagram of the setup and a schematic representation of the system with the cooled coil containing a sample inside.

**Figure 8:**
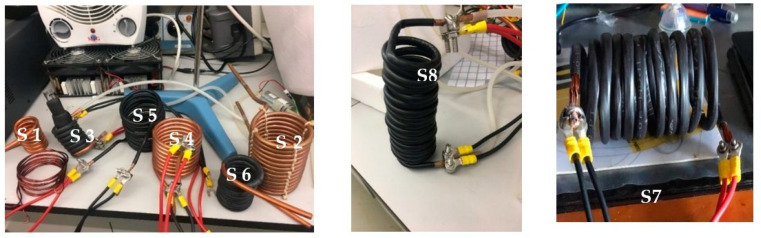
Images of solenoids described in Table 4.

To improve the performance of the magnetic hyperthermia system, various experiments were conducted with different coil configurations (see Table 4) that are depicted in Figure 8. The results of these experiments will be presented throughout the text.

### 3.2. Results of Magnetic Field Simulations 

This section presents the numerical results obtained for the different coils used in the hyperthermia studies described in this work.

Parametric frequency studies of solenoid coils

**Figure 9:**
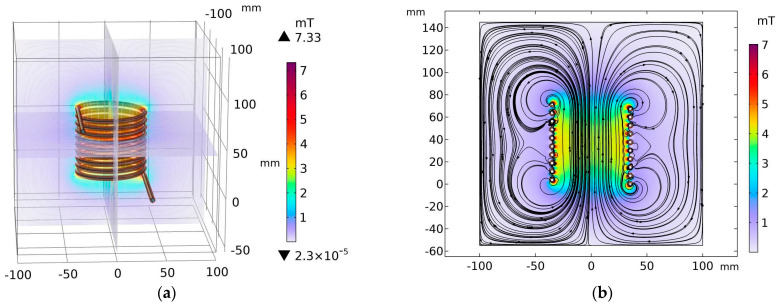
Profile of the magnetic field density (mT) of solenoid coil S5 for a driving voltage of 60 V and an operating frequency of 72 kHz. (**a**) Three-dimensional view, (**b**) two-dimensional view for the plane x = 0.

The geometric parameters of each solenoid coil can be found in Table 4. In these numerical studies, only coils S5, S6, and S8 were simulated (see example of S5 in Figure 9), for reasons detailed in the next sub-section. For coil S8, two simulations were conducted with different numbers of turns, 12 and 17. To obtain the electric properties of the selected coils, a parametric frequency run was performed using frequencies of 72, 96, 134, and 302 kHz. The applied voltage to excite the coils was set to a constant value of 60 V.

Comparison of coils at various frequencies

To compare each coil, two criteria were established: the shape of the magnetic field density at the center of the coil and the electric properties of each coil. The first criterion involved evaluating the magnetic field density relative to the coil’s length. This method allowed us to determine whether the magnetic field density inside the coil followed a horseshoe profile or remained constant along its length. The second criterion focused on the electrical properties, such as impedance, current, and induction, when different frequencies were applied to the solenoid.

The initial solenoids (S1–S4) were tested but did not yield significant results; therefore, simulations were only performed for S5–S8. Solenoid S1 did not produce a satisfactory magnetic field value and, like S2, exhibited a less concentrated, non-uniform field with high impedance. S3 presented challenges in implementing an insulation system due to its narrow design, while S4 had a less concentrated magnetic field compared to S2. Additionally, coil S7 was discarded because it heated up as the electric current passed through it.

**Figure 10:**
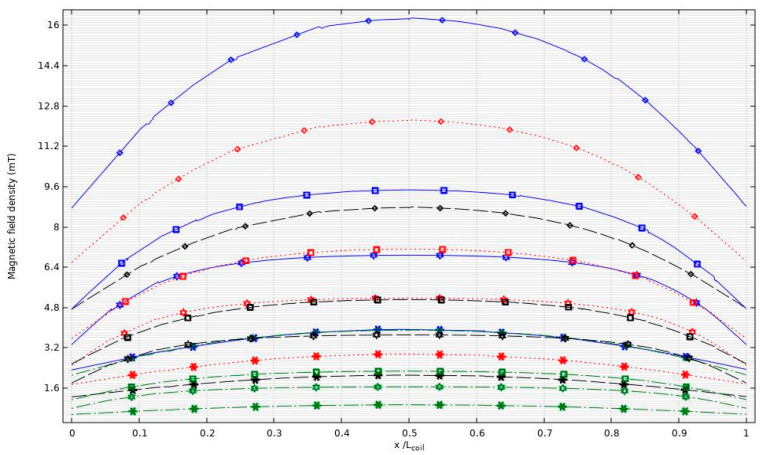
Magnetic field density (mT) along the center of the simulated coils at various operating frequencies.

Figure 10 displays the results of the first criterion evaluation. The blue solid lines represent coils operating at a frequency of 72 kHz, while the red dotted lines, black dashed lines, and green dashed–dot lines correspond to frequencies of 96 kHz, 134 kHz, and 302 kHz, respectively. The symbols (*), (◊), (□), and (☼) denote solenoid coils S5, S6, S8 with 12 turns, and S8 with 17 turns, respectively.

Analysis of the image reveals that the magnetic field strength decreases with increasing frequency, as demonstrated by [40]. Overall, coil S6 exhibited the highest magnetic field density, reaching peaks above 16 mT at 72 kHz and approximately 3.5 mT at 302 kHz. In contrast, coil S5 had the lowest value, peaking at around 3.5 mT at 72 kHz and below 1 mT at 302 kHz. However, coil S8, both with 12 turns and 17 turns, demonstrated a consistent magnetic field strength across all frequencies, showing a flat profile.

The second criterion analysis is based on Table 5, which presents the electrical properties of each solenoid excited by a sinusoidal applied voltage of 60 V. As shown, S6 exhibited the highest current values and consequently the lowest impedance inductance, ranging from 1.07 μH to 1.08 μH. S8 achieved impedances ranging from 2.13 μH to 2.15 μH for 12 turns and from 3.03 μH to 3.06 μH for 17 turns. Similar to the trend observed in the first criterion evaluation, S5 demonstrated the poorest performance, with inductances ranging between 3.91 μH and 3.93 μH. This is consistent with Equation (10), as the impedance varies linearly with the inductance of the coil and the frequency. Additionally, Equation (11) describes the inductance, indicating a quadratic relationship with the number of turns and an inverse linear relationship with the length of the coil. Thus, the simulation results align with the expected outcomes.

### 3.3. Hyperthermia Essays 

**Figure 11:**
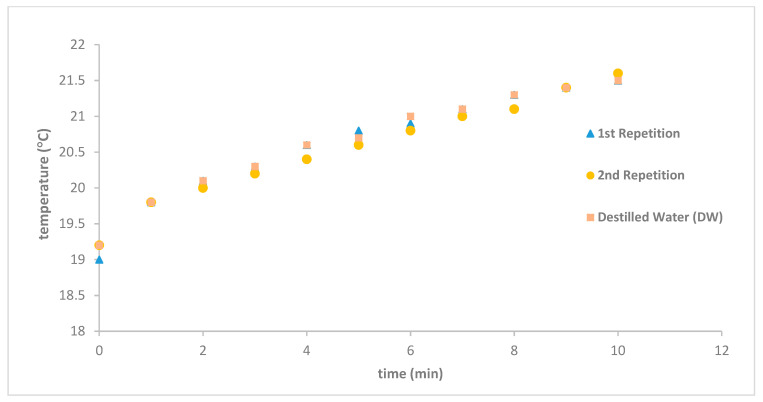
The temperature variation of dstillated water samples was consistent across three experiments conducted on different days at a frequency of 69 kHz with solenoid S5.

In order to assess the effectiveness of the insulation system, a critical component for adiabatic heating, we conducted tests using distilled water (DW) and observed its heating patterns. As depicted in Figure 11, over a 10 min heating period at a frequency of 69 kHz, the temperature rose by a mere 2.5 °C across three separate days, affirming the consistency of our measurements. Initially, we meticulously monitored the sample’s temperature on a minute-by-minute basis to ascertain any signs of heating. Once the device’s reliability was confirmed, we obtained continuous data and meticulously documented it for the purpose of calculating temperature variation per second. This approach enabled us to utilize more precise methodologies, such as the Lucas box method and the increment-corrected method, to derive the SAR value [39,41].

**Figure 12:**
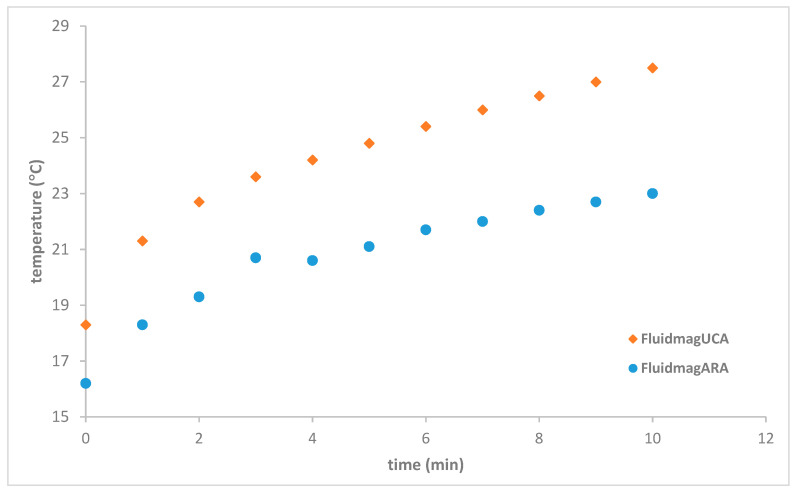
Heating curves for the Fluidmag_ARA_ and Fluidmag_UCA_ samples at a frequency 98 kHz using solenoid S5.

The initial experiments were conducted using solenoid S5, and the first particles tested were Fluidmag_ARA_ and Fluidmag_UCA_. Fluidmag_ARA_ consists of coated particles, while Fluidmag_UCA_ features uncoated particles with anionic charges. The nanoparticle concentration used was 25 mg/mL. Fluidmag_ARA_ particles are composed of a multi-domain magnetite core coated with a layer of glucuronic acid [29,42]. At a frequency of 98 kHz, the temperature increased by 9.2 °C in the Fluidmag_UCA_ sample and 6.8 °C in the Fluidmag_ARA_ sample. These results align with the parameters listed in Table 5, as Fluidmag_ARA_ particles are smaller than UCA particles, resulting in fewer less magnetic domains and a lower iron percentage. Additionally, it is known that the coating on nanoparticles can reduce the magnetic saturation value (Ms), which is consistent with the observed results. The heating curves and SAR values are presented in Figure 12, showing SAR values of 1.76 W/g_MNP-UCA_ and 1.37 W/g_MNP-ARA_.

**Figure 13:**
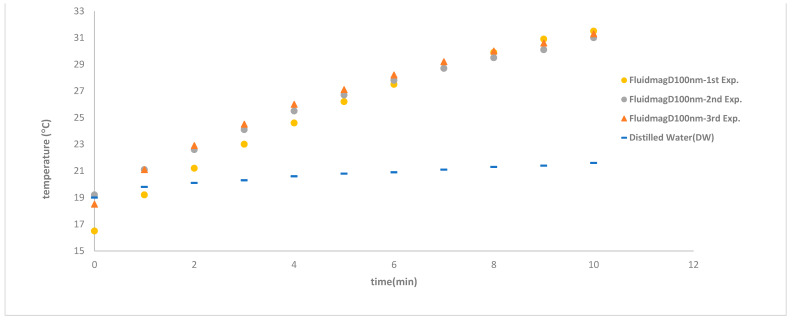
Heating curves of Fluidmag_D100nm_ (25 mg/mL) at 69 kHz using solenoid S5.

To verify that the heating was specifically due to the presence of the magnetic particles, we conducted a control experiment using a blank sample of distilled water (DW) with the same volume as the analyzed samples. The heating curve for this blank sample showed a temperature increase of only 2 °C. Additionally, we studied a sample of FluidMagD100nn at a frequency of 69 kHz and repeated the experiment at least three times to assess its reproducibility. The resulting specific absorption rate (SAR) was 4.34 W/gMNP (69 kHz), and the system and procedure exhibited excellent reproducibility, consistently reaching nearly the same final temperature within the last 10 min, even when starting at a lower temperature. The higher SAR values observed align with those reported by Kallumadil and colleagues [30]. In Figure 13, it can be seen that the temperature did not reach the expected plateau associated with magnetic hyperthermia, where a stable equilibrium between heat lost and heat generated is achieved. The main aim of this study was to confirm that the heating was indeed caused by the magnetic nanoparticles and to validate that the device operates correctly with acceptable reproducibility. This was confirmed by the third repetition: although the initial temperature was lower, the final temperature was almost the same in the last 10 min. Given more time, the final temperature would likely have reached a stable state similar to previously observed results.

**Figure 14:**
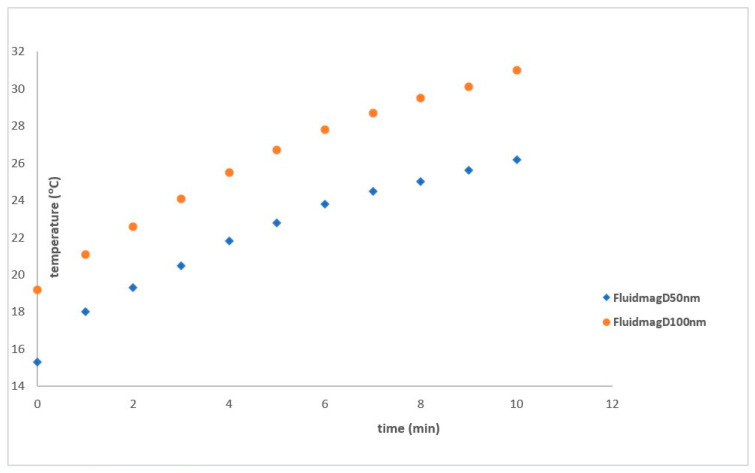
Heating curves of Fluidmag_D50nm_ (25 mg/mL) at 69 kHz using solenoid S5.

Figure 14 illustrates the heating curve of FluidmagD50 at 69 kHz using solenoid S5. The SAR values obtained were 2.47 W/g_MNP_ for FluidmagD50 and 3.5 W/g_MNP_ for FluidmagD100. Even though distilled water samples were used in all experiments, their results are not displayed in the graphs to avoid redundancy.

**Figure 15:**
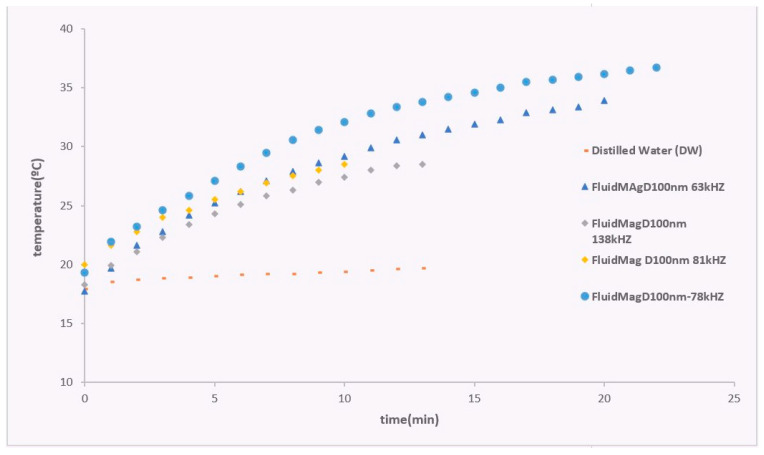
Heating curves for the FluidMag_D100nm_ sample at various frequencies using solenoid S5.

The study investigated how frequency impacts the heating curves of the samples to find the best frequency. Figure 15 displays the linear fitting that was utilized to find the initial slope for calculating the SAR value, as outlined in Equation (9).

The slight variation in frequency, ranging from 63 to 138 kHz, leads to a corresponding change in the final temperature. This change is due to the differences in the current magnitude at different frequencies. At 63 kHz, the current is higher compared to 138 kHz due to impedance. To address this issue, it is necessary to either increase the voltage of the power source or design a new coil. The frequency of 78 kHz produces the best results, as it is higher than 63 kHz but still maintains a similar current magnitude.

**Figure 16:**
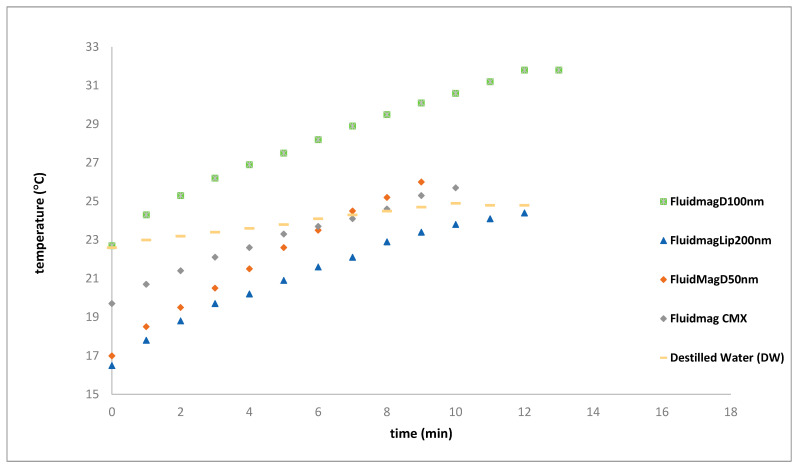
Heating curves of various samples at a frequency of 138 kHz.

The SAR values obtained are as follows: 2.43 W/g_MNP_ at 63 kHz, 2.22 W/g_MNP_ at 81 kHz, 3.54 W/g_MNP_ at 78 kHz, and 2.03 W/g_MNP_ at 138 kHz. Since the magnetic field is directly proportional to the current, as described in Equation (4), increasing the current significantly impacts this parameter. It is possible that if we had continued the experiment at 138 kHz, the temperature would reach a value like what was observed at 13 min at 78 kHz. In the case of the FluidMag_Dx100nn_ sample, a final temperature of 36.7 °C was reached at 78 kHz using the system configuration with the refrigerated solenoid S5. This produced a SAR value of 3.54 W/_gMNP_ (refer to Figure 15). These results are promising, as they were achieved using a lower frequency of 78 kHz and a lower magnetic field compared to the study by Kallumadil and colleagues. Furthermore, experiments were carried out with various types of particles to compare heating curves at 138 kHz and to determine the optimal system for heating studies. The SAR values obtained were as follows: FluidMag_CMX_ = 1.56 W/gMNP, FluidMag_Lip200nm_ = 1.92 W/g_MNP_, FluidMag_Dx100nn_ = 2.03 W/g_MNP,_ and FluidMag_Dx50nm_ = 2.70 W/g_MNP_ (refer to Figure 16).

Figure 16 summarizes the results obtained for the four samples at 138 kHz. Interestingly, the FluidMag_200 nm_ sample seems to exhibit lower heating efficiency, whereas the FluidMag_CMX_ sample, with a broader size distribution as reported by the manufacturer, demonstrates a less intense heating effect. This difference may be attributed to magnetic properties, such as higher magnetization values associated with larger MNPs.

Furthermore, a new set of experiments employing a new coil, designated as S6, with reduced turns, and narrower and shorter dimensions, was performed. Given that the inductive reactance $\;X_{L}$ relies on inductance (L) and the length of the solenoid $l_{e}$, as outlined in Equations (10) and (11), it is anticipated that the alterations in the coil design will impact the heating performance.

(10) $$X_{L}=2\times \pi \times f\times L\;\;\;[\mathrm{ohms}]$$

(11) $$L=\frac{\left(N^{2}\times A\times \mu \right)}{l_{e}}\;\;\;[\mathrm{Henry}]$$

**Figure 17:**
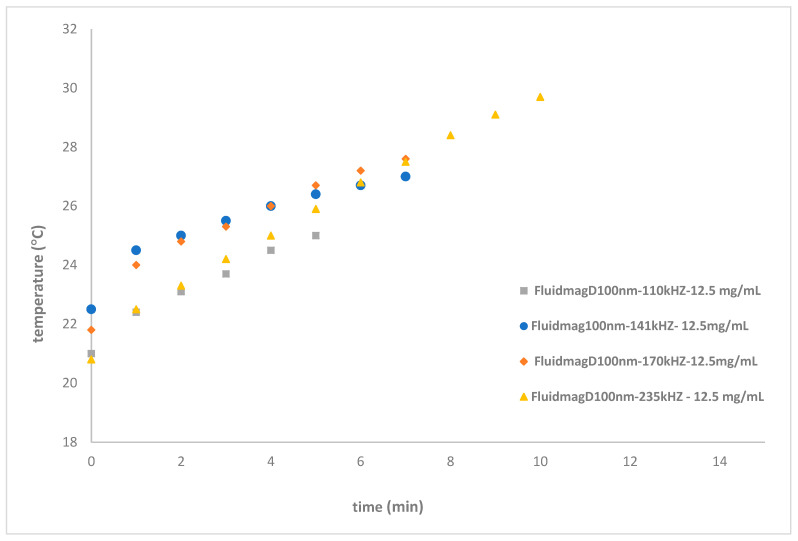
Heating curves of FluidMag_Dx100nn_ at different frequencies with a concentration of 12.5 mg_MNP_/mL using solenoid S6.

In Equation (10), the symbol (f) represents the frequency, L denotes the inductance of the solenoid, N is the number of turns, µ is the modulus of the magnetic permeability of the material, A is the cross-sectional area in m^2^, and $l_{e}$ is the length of the solenoid. The objective was to reduce the length of the solenoid, thereby decreasing $l_{e}$, and to concentrate the magnetic field by using a narrower inductor and a reduced cross-sectional area with the new coil, S6. The FluidMag_Dx100nm_ sample, with a concentration of 12.5 mg_MNP_/mL, was tested to assess improvements in heating performance. The results showed final temperatures ranging from 28.8 °C to 29.7 °C on the frequencies 110–170 kHz. The corresponding SAR values were 2.62 W/g_MNP_ at 141 kHz, 3.60 W/g_MNP_ at 110 kHz, and 3.49 W/g_MNP_ at 170 kHz (see Figure 17).

**Figure 18:**
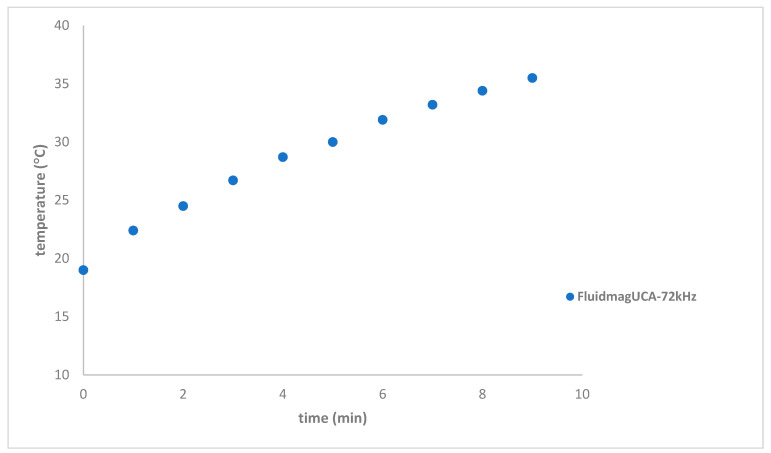
Heating curves of FluidMag_UCA_ at 72 kHz with a concentration of 12.5 mg_MNP_/mL using solenoid S8.

A new coil configuration, designated as S8, was designed with more turns and a smaller radius, resulting in a more concentrated magnetic field. This configuration improved system performance, achieving an alternating magnetic field (AMF) H = 4 ±2 mT. The heating of the Fluidmag_UCA_ sample, with a concentration of 12.5 mg/mL, improved, reaching 35.5 °C in 9 min at 72 kHz, with a SAR of 4.83 W/g_MNP UCA_ (see Figure 18). For the FluidMag_D100nm_ sample, the SAR was 3.1 W/g_MNP_, which is consistent with the literature values [43].

These results can be explained by the S8 coil’s 17 turns and smaller diameter, which concentrate the magnetic field lines. Additionally, the current passing through the solenoid is nearly doubled (see Table 5), which is directly proportional to the intensity of the alternating magnetic field.

**Figure 19:**
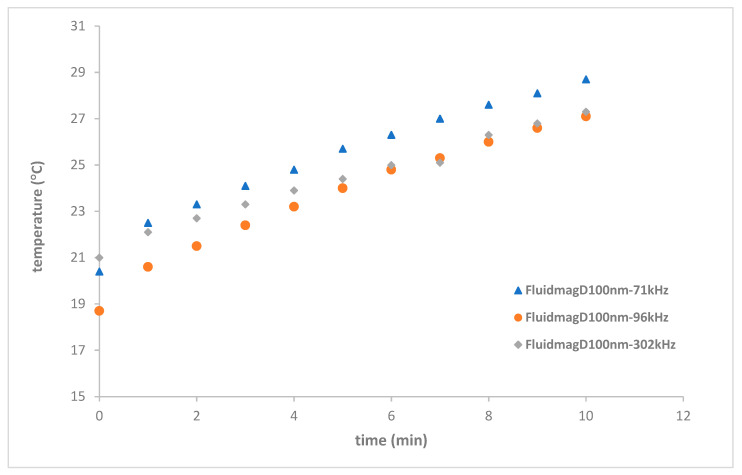
Heating curves of FluidMag_D100nm_ at different frequencies with a concentration of 12.5 mg_MNP_/mL using solenoid S8.

Regarding the MagFluid_D100nm_, the previous results were significantly affected, reinforcing the notion that the maximum temperature threshold was likely reached within the frequency range previously observed with coil S6 (see Figure 19).

**Figure 20:**
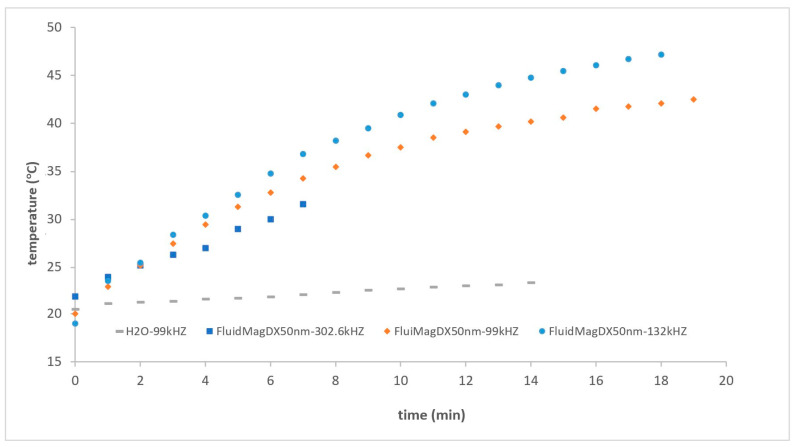
Heating curves of FluidMag_Dx50nm_ at different frequencies with a concentration of 25 mg_MNP_/mL using solenoid S8.

FluidMag_Dx50nm_ samples were used at different frequencies with a concentration of 25 mg_MNP_/mL in this new device configuration. As anticipated, the results improved, and the graph also shows the reference for the DW sample and the repeatability in this new configuration (see Figure 20). The SAR values were as follows: 3.55 W/g_MNP_ at 302 kHz, 5.40 W/g_MNP_ at 99 kHz, and 7.74 W/g_MNP_ at 132 kHz. The optimal frequency was around 132 kHz. The Chemicell samples exhibit rapid heating under the 132 kHz applied magnetic field, consistent with the findings of Eggeman and co-workers [44], with the temperature notably increase at 132 kHz.

**Figure 21:**
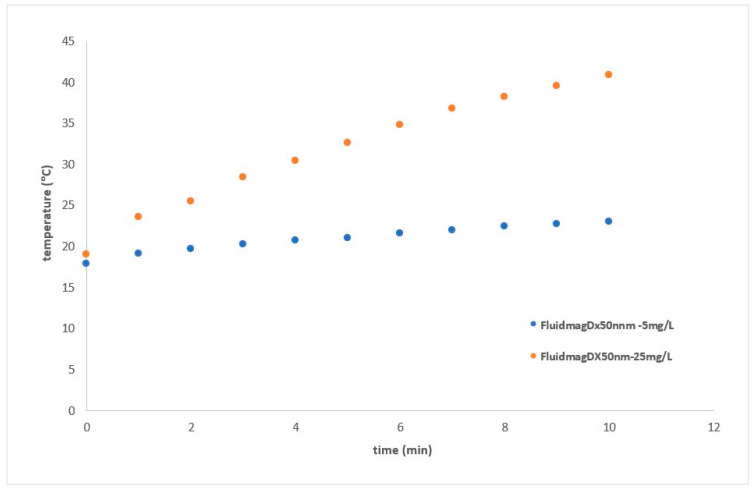
Heating curves of FluidmagDX_50nm_ samples with different concentrations (5 and 25 mg/m_lMNP_) at 132 kHz using solenoid S8.

The heating curves for the two samples of FluidMag_DX50nm_ are shown in Figure 21, as a function of average concentration (5–25 mg/mL). The Chemicell samples were heated using an applied magnetic field at 132 kHz, with a field amplitude of approximately 8 mT. The initial slope decreases when the sample is diluted from 25 mg/mL to 5 mg/mL, which aligns with the findings of [44]. Therefore, it appears that dilution does not improve the heating efficiency in this type of MNPs. As state previously, macroscopic dilution does not necessarily change the inter-particle distance within a cluster; it may only increase the average cluster spacing, as the MNPs are composed of clusters as described before.

At a frequency of 132 kHz, FluidMagDX50nm exhibits a specific absorption rate (SAR) of 1.95 W/g for a sample concentration of 5 mg/mL and 9.13 W/g for a sample concentration of 25 mg/mL. The final temperature for the more concentrated sample reached 47.2 °C.

**Figure 22:**
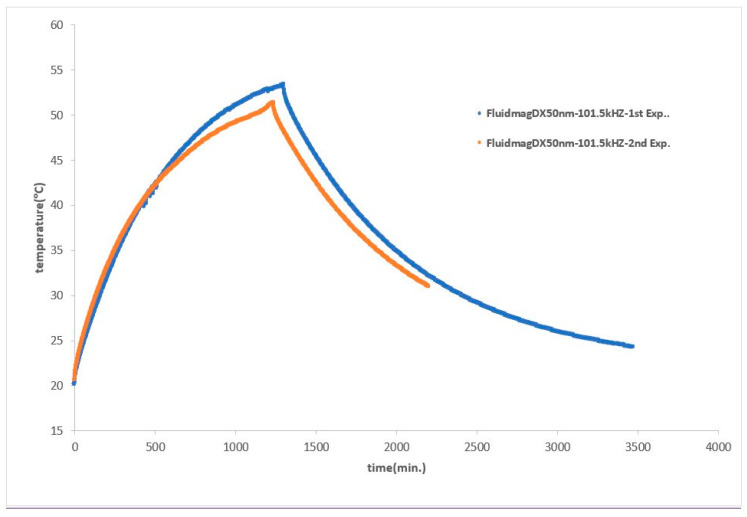
Heating and cooling curves of FluidMagDx50nm with the final configuration (S8).

To summarize, we tested the FluidMag_DX50nm_ sample at 101.5 kHz using a new configuration where we changed the capacitors to a specific value determined by us through Equation (7) to achieve a higher frequency. We selected these particles because they have previously produced good results [30]. Therefore, we wanted to determine if our device could achieve comparable or even better results than those obtained by other researchers. We conducted a comprehensive experiment for magnetic hyperthermia, which included heating and cooling curves. The results were very promising, as depicted in Figure 22, yielding a SAR of 10.8 W/gMNP.

A resume of the final temperature and results obtained from all experiences are presented in Table 6 with the respective average and deviation; almost all of the experiences were performed in triplicate as a minimum.

## 4. Conclusions

In this study, we developed, built, and tested a new magnetic hyperthermia device. We evaluated its performance using commercial magnetic nanoparticles and found that it produced results similar to those of commercial devices. We optimized the system to achieve higher magnetic fields and frequencies, and the simulation results confirmed our practical findings. This device is more cost-effective and portable compared to existing options, and we aim to make this technology more accessible.

According to our findings and previous research [25,26,45], the Chemicell iron oxide sample FluidMagDx appears to be a suitable material for magnetic hyperthermia (MHT) applications. The cluster size is large enough to prevent endocytosis at a cell membrane, yet small enough to avoid activating bacterial defense mechanisms. The coating material, dextran, makes it biocompatible. However, the stability of the clusters inside biological organisms is still unknown [46]. As a result, “FluidMAG” from Chemicell GmbH showed a significant heating response that can be adjusted by the field and frequency of the AC driving field and the solution concentration.

Our results confirm that significant optimization can be achieved in a magnetic hyperthermia device, such as increasing the frequency, the magnitude of the magnetic field, or even designing a programmable system.

According to our results, factors such as the size, composition, and shape of particles may not be enough to accurately predict the magnetic response to different frequencies and, as a result, the rate of heat generation in an AC magnetic field. We can only make initial assumptions based on previous research, so our next step is to create various types of magnetic nanoparticles and see how they affect the heating rate.

The concentration of iron in the sample influences the heating, but local clustering is also important.

In our upcoming research, we will concentrate on creating MNPs and adjusting their properties to improve their performance. We are in the process of designing and building a new apparatus that is similar to the resonant system but capable of generating higher magnetic fields and frequencies, as outlined in Castelo-Grande et al. [39].

We have encountered an issue with controlling test frequencies and have addressed it by implementing a circuit based on the H-bridge using MOSFET transistors (IRFP260 or IRFP4668) as switches. These switches are controlled by a computational system (Arduino MEGA platform), enabling us to generate square waves at the desired frequency. However, we faced challenges in controlling the switches on the upper part of the H-bridge (H-bridge high side or low side).

Based on our analysis, it is evident that there is still significant room for improvement in the field of magnetic nanoparticle hyperthermia. This could involve developing more user-friendly devices with a wider range of designs and configurations, thereby paving the way for further exploration. In terms of MNPs, there is still much to learn in order to accurately predict the optimal frequency and field for MH, which are closely linked to intrinsic parameters [47].

For possible future developments, including for example, ex vivo and in vivo biological sample applications, and comparison with simulated data (on the hyperthermia treatment), collaborations with health institutes of Porto and Salamanca have been initiated.

## 5. Patents

A patent (reference PPP 20242006604014) was filed concerning the developed innovative magnetic hyperthermia device described in this paper.

## Figures and Tables

**Table 1:** Advantages and disadvantages of magnetic hyperthermia [14,15,16,17,18].

Advantages	Disadvantages
Magnetic nanoparticles are absorbed by cancer cells, allowing for localized therapeutic heat supply, which increases hyperthermia’s effectiveness	Magnetic hyperthermia encounters challenges in enhancing nanoparticle heating power, regulating tumor temperature
Tagging MNPs with tumor-specific binding agents ensures targeted and efficient treatment, maximizing its impact.	It is difficult to minimize adverse effects on nearby healthy tissues.
Harmless passage of alternating magnetic field frequencies through the body exclusively generates heat in MNP-containing tissues, ensuring safe and precise treatment.	It is necessary to monitor temperature changes at the cellular level using precise and non-invasive techniques.
MNPs’ ability to traverse the blood–brain barrier makes them valuable for treating brain tumors.	It is crucial to understand the impact of temperature on the biological processes of cells.
MNPs can be used to create stable colloids, allowing for a variety of drug delivery routes.	It is important to comprehend the factors that impact heat transport from MNPs to cells.

**Table 2:** Properties for magnetostatic simulations.

Material	Relative Permeability —$\mu_{r}$	Relative Permeability —$\varepsilon_{r}$	Electric Conductivity —σ (S/m)
Air	1	1	0
Copper	1	1	6 × 10^7^
Water	1	1	0

**Table 3:** Properties of the magnetic nanoparticles used in the experiments.

Product	Matrix/Cover	Size (Hydrodynamic Diameter) (nm)	Density (g/cm^3^)	Functionalization	Particles Number	Core/vh * (nm)	Zeta Potential (mv)
Fluidmag-ara	Polysaccharide	150	~1.25	Glucuronic acid	~1.8 × 10^15^/g −2.2 × 10^14^/g	23.6 ± 5.6/ 121 ± 40 [30]	−22.65 mV [30]
Fluidmag-uca	No	200	~5.2	Anionic charge	~2.2 × 10^14^/g		
Fluidmag-dx	Dextran	50	~1.25	Hydroxyl groups	~1.3 × 10^16^/g		
Fluidmag-cmx	Carboxymethyl–dextran	n.a.	~1.25	Sodium carboxylate		110.6 ± 3.5 [32]	–32.3 ± 0.1
Fluidmag-lipid	Phosphatidylcholine	200	~1.25	Phosphatidylcholine	~2.2 × 10^14^/g		
Fluidmag-d50	Starch	50	~1.25	Hydroxyl groups	~1.3 × 10^16^/g		
Fluidmag-d100	Starch	100	~1.25	Hydroxyl groups	~1.8 × 10^15^/g		

Supermagnetic_ARA_, coated with polysaccharides, distilled water solvent (diam. = 150 nnm, conc = 25 mg/mL and 1.8 × 10^15^ particles/g; d = 1.25 g/cm^3^). Supermagnetic_UCA_ (diam. = 200 nm, conc = 25 mg/mL and 2.2 × 10^14^ particles/g; d = 5.2 g/cm^3^), magnetite as core and uncoated, and are charged to be anionic, solvent distilled water. * Other average values published CMX-coated_MNPs_ 0.98 ± 0.11 content of iron mg_Fe_/mL.

**Table 4:** Parameters of the applied solenoids.

Solenoid	H (Height) (mm)	Tube External Diameter (mm)	Tube Internal Diameter (mm)	Solenoid Internal Diameter (mm)	Number of Turns	Space Between Turns (mm)	Observations
S1	33.0	4.1	----	54.6	5	2.7	
S2	100.7	6.3	1	73.7	13	2.0	Inductance is 5 μH
S3	68.0	6.3	1	50.0	9	2.0	
S4	66.0	6.3	1	74.5	8	Variable	
S5	73.0	5.0	1	70.0	9	2–2.5 variable	
S6	57.0	6.3	1	41.3	7	Variable	Inductance is 2.5 μH
S7	100.0	n.a.	---	70.0	11	Variable	Foldable (multiwire)
S8	119.6	5.0	1	42.3	17	Variable	Yield the best results so far

**Table 5:** Electrical properties of solenoid at various frequencies.

	f(kHz)	Coil Current (A)	Coil Resistance (Ω)	Coil Impedance (Ω)	Coil Inductance (μH)
S5	72	33.684∠−89.305°	2.160 × 10^−2^	1.781∠89.305°	3.9372
	96	25.311∠−89.403°	2.470 × 10^−2^	2.37∠89.403°	3.9296
	134	18.167∠−89.512°	2.811 × 10^−2^	3.303∠89.512°	3.9226
	302	8.081∠−89.739°	3.377 × 10^−2^	7.425∠89.739°	3.9127
S6	72	122.183∠−88.89°	9.512 × 10^−3^	0.491∠88.89°	1.0853
	96	91.917∠−89.046°	1.086 × 10^−2^	0.653∠89.046°	1.0820
	134	66.04∠−89.22°	1.237 × 10^−2^	0.909∠89.22°	1.0790
	302	29.423∠−89.58°	1.495 × 10^−2^	2.039∠89.58°	1.0747
S8—12 turns	72	61.764∠−88.943°	1.791 × 10^−2^	0.971∠88.943°	2.1470
	96	46.459∠−89.101°	2.026 × 10^−2^	1.291∠89.101°	2.1408
	134	33.372∠−89.277°	2.268 × 10^−2^	1.798∠89.277°	2.1353
	302	14.858∠−89.626°	2.637 × 10^−2^	4.038∠89.626°	2.1280
S8—17 turns	72	43.316∠−88.959°	2.516 × 10^−2^	1.385∠88.959°	3.0614
	96	32.58∠−89.116°	2.841 × 10^−2^	1.842∠89.116°	3.0528
	134	23.402∠−89.29°	3.178 × 10^−2^	2.564∠89.29°	3.0450
	302	10.418∠−89.634°	3.676 × 10^−2^	5.759∠89.634°	3.0349

**Table 6:** Results of the final temperature respective deviations and conditions obtained in the experiences.

Type of Particle	Frequency (kHz)	Solenoid	T_Final_ (C°)	Figure
Fluidmag_ARA_	98	S5	27.5 ± 0.4	12
Fluidmag_UCA_	98	S5	23.5 ± 0.5	12
FluidMag_D100nm_	69	S5	31.0 ± 0.5	13
FluidMag_D50nm_	69	S5	26.2 ± 0.5	14
FluidMag_D100nm_	69	S5	31.5 ± 0.4	14
FluidMag_D100nm_	63	S5	31.0 ± 0.3	15
FluidMag_D100nm_	78	S5	33.8 ± 0.3	15
FluidMag_D100nm_	81	S5	25.5 ± 0.4 ^+^	15
FluidMag_D100nm_	138	S5	28.5 ± 0.4 ^++^	15
FluidMag_CMX_	138	S5	27.7 ± 0.5 *	16
FluidMag_Lip200nm_	138	S5	24.4 ± 0.4 **	16
FluidMag_Dx100nn_	138	S5	31.80.5 ^+^*	16
FluidMag_DX50nm_	138	S5	26.9 ± 0.5 ^+^**	16
FluidMag_Dx100nn_^a^	110	S6	27.0 ± 0.5	17
FluidMag_Dx100nn_^a^	141	S6	27.0 ± 0.4	17
FluidMag_Dx100nn_^a^	170	S6	27.5 ± 0.4	17
FluidMag_Dx100nn_^a^	235	S6	29.7 ± 0.3	17
FluidMag_UCA_^a^	72	S8	35.5 ± 0.4	18
FluidMag_Dx100nm_	71	S8	28.7 ± 0.4	19
FluidMag_Dx100nm_	96	S8	27.1 ± 0.5	19
FluidMag_Dx100nm_	302	S8	27.3 ± 0.5	19
FluidMag_Dx50nm_	99	S8	34.3 ± 0.4	20
FluidMag_Dx50nm_	132	S8	36.8 ± 0.4	20
FluidMag_Dx50nm_	302	S8	31.6 ± 0.4 ^++^**	20
FluidMag_Dx50nm_^b^	132	S8	23 ± 0.3	21
FluidMag_Dx50nm_	132	S8	40.9 ± 0.	21
FluidMag_D50nm_	101.5 kHz	S8	52.2 ± 0.4	22

^+^ At 10 min, ^++^ at 13 min.; * 10 min; ** 12 min; +* 13 min; +** 9 min; ^a^ concentration of 12.5 mgMNP/mL ^b^ concentration of 5 mg/mL; ^++^** The study was stopped for 302 kHz 31.6 in 7 min since at the same time it obtained 36.8 (132 kHz) and 34.3 (99 kHz); ^b^ 5 mg/mL.

## Data Availability

Data are contained within the article.

## Appendix A

*Magnetic field measurement*

The LF-R 400 H field is a passive near-field probe that has a large diameter (25 mm), making it highly sensitive and suitable for measurements in ranges of up to 10 cm around assemblies and fixtures, as it is proven capable of detecting a larger area of the magnetic field specially comparing with the LF-R 50 (10 mm) or LF-R 3 (3 mm) near-field probes). It has a current attenuation sheath and is, therefore, electrically shielded. It can be connected to a spectrum analyzer or an oscilloscope with a 50 Ω input. An oscilloscope with a 1:1 tip was used, which recorded the V_value_ (peak to peak) and verified the sinusoidal waveform, as well as the frequency obtained in the coil.

*Equations used to calculate the magnetic field*

Probe RF-R 400-1- Langer EMV-Technik:

(A1) $$\lceil dB\rceil ==\mu \left[dB_{\mu }\right]\frac{A}{m}=U_{probe}\left[dB_{\mu }V\right]+\left[dB\frac{\mu A}{\mu Vvm}\right]$$

A signal attenuator was also used to be able to read the obtained signal. For this we have to convert the value read by the probe with the attenuator. Since this is 5 dB, the following equation must be solved:

(A2) $$5\left(dB\right)=20\times log_{10}\left(x\right)\;\;\;\;\;\;\;\mathrm{where}\;\mathrm{we}\;\mathrm{want}\;\mathrm{to}\;\mathrm{obtain}\;x$$

Solving, we obtain the value of x = 0.56, which means it attenuates 56% of the value.

(A3) $$or\;5\left(dB\right)=20\times log_{10}\left(\frac{V_{out}}{V_{in}}\right)\;where\;we\;want\;to\;obtain\;v_{in}$$

For example, if the value read was [∆V]_pp_ = 95 ± 5 V <=>95/0.56 = 169.64, which gives approximation—[∆V]_p_ = 85, then it is necessary to resolve the following equation in order to obtain AMF.

(A4) $$H\left(\frac{kA}{m}\right)=ref_{value\left(kHz\right)}\times 10^{\left(\frac{V_{p}}{20}\right)/1000}$$

where $ref_{value\left(kHz\right)}$ is a value obtained from the data sheet of the equipment and it is the function of frequency used, $V_{p}$ value of the high of pic of the wavelength shown in the oscilloscope. In the particular case of this work, the range of magnetic field is from 3.8–6.5 kA/m or 4.6–8 mT depending on the configuration system (different solenoids).
